# An enhanced CNN-LSTM remaining useful life prediction model for aircraft engine with attention mechanism

**DOI:** 10.7717/peerj-cs.1084

**Published:** 2022-08-30

**Authors:** Hao Li, Zhuojian Wang, Zhe Li

**Affiliations:** 1 Air Force Engineering University, Graduate School, Xi’an, Shaanxi, China; 2 Air Force Engineering University, Aeronautics Engineering College, Xi’an, Shaanxi, China

**Keywords:** Aircraft engine, Remaining useful life, Convolutional block attention module, Convolutional neural network, LSTM

## Abstract

Remaining useful life (RUL) prediction is one of the key technologies of aircraft prognosis and health management (PHM) which could provide better maintenance decisions. In order to improve the accuracy of aircraft engine RUL prediction under real flight conditions and better meet the needs of PHM system, we put forward an improved CNN-LSTM model based on the convolutional block attention module (CBAM). First, the features of aircraft engine operation data are extracted by multi-layer CNN network, and then the attention mechanism is processed by CBAM in channel and spatial dimensions to find key variables related to RUL. Finally, the hidden relationship between features and service time is learned by LSTM and the predicted RUL is output. Experiments were conducted using C-MPASS dataset. Experimental results indicate that our prediction model has feasibility. Compared with other state-of-the-art methods, the RMSE of our method decreased by 17.4%, and the score of the prediction model was improved by 25.9%.

## Introduction

The aircraft engine is one of the core equipment of aircraft, which largely determines the flight performance and flight safety of aircraft. With the development of maintenance theory, sensing and communication technology, aircraft prognosis and health management (PHM) is increasingly a significant way to improve the reliability and safety of aircraft (Li, Verhagen & Curran, 2020). PHM collects operational data in real time through sensors, and realizes the functions of aircraft condition monitoring, fault prediction and maintenance planning, among which the remaining useful life(RUL) prediction is a vital part of PHM system (Che et al., 2019).

At present, the RUL prediction methods can be roughly divided into two categories. They are methods based on the physical model and methods based on monitoring data (Peng, Ye & Chen, 2019; Singh et al., 2020; Ren et al., 2018; Shankar kumar, Kumaraswamidhas & Laha, 2021). The physical model-based method is usually for constructing and estimating the degradation model of equipment mathematically by summarizing the rules of failure mechanism (Lei et al., 2018). This method is more suitable for the crack propagation of metal components, *etc*. Ye et al. (2018) studied a fatigue life prediction method based on multi-scale crack propagation model, and the prediction ability of this method was verified by nickel-based GH4169 alloy. However, for complex systems and equipment, the method based on physical model is no longer applicable, mainly because in industrial practice, we don’t fully grasp the failure mechanism of equipment, and the process of establishing mathematical model is very complicated. Therefore, more studies on RUL prediction mainly focus on data-based methods, which could be divided into three categories (Muneer et al., 2021; Sun et al., 2021; Shankar kumar, Kumaraswamidhas & Laha, 2021). One is to describe the degradation of equipment through stochastic process. Gao et al. (2019) studied the characteristics of light-emitting diode driving power, and proposed a RUL prediction method based on Wiener process. Lin et al. (2021) proposed a novel method to model two-phase degenerate behavior of products based on the nonlinear Wiener process. The second is to establish RUL prediction model by machine learning. Ordez et al. (2019) proposed a RUL prediction model which combined support vector regression model (SVM) and auto-regressive integrated moving average (ARIMA) model. Pan et al. (2020) put forward a two-stage prediction method based on extreme learning machine (ELM). Their method could quickly and accurately predict the RUL of rolling bearings. Kamat, Sugandhi & Kumar (2021) used the unsupervised machine learning for anomaly trend analysis of bearings, and the semi-supervised method for RUL prediction. The third category is the deep learning prediction method that has emerged with the development of artificial intelligence. Wen et al. (2021) established a deep learning architecture based on bidirectional gated recurrent unit (BGRU), and proposed a domain-adaptive RUL prediction model. Guo et al. (2017), Kim & Liu (2021) and Khumprom, Grewell & Yodo (2020), respectively use three network models, deep belief network (DBN), bayesian deep learning framework, and feedforward neural network(FNN) to establish different RUL prediction models, and all of them achieved good prediction results.

Aircraft engine monitoring data has the features of large amount of data and high dimensionality, and deep learning has better feature extraction ability in processing such data (Mao et al., 2022; Zhao & Wang, 2021), so the prediction method of deep learning is more suitable for aircraft engine RUL prediction. At present, some scholars have also conducted relevant research. Hu et al. (2021) built several new DBRNNs with new network configurations and designed a new customized loss function for RUL prediction of aircraft engine. Chui, Gupta & Vasant (2021) proposed a RUL prediction algorithm in combination with RNN and LSTM, used non-dominated sorting genetic algorithm II (NSGA-II) to optimize it, and achieved better results. Li et al. (2021) proposed a new Bayesian deep learning nework considering the epistemic uncertainty and aleatoric uncertainty to solve the prognostic uncertainty problem. Ragab et al. (2021) addressed the domain shift problem based on contrastive adversarial domain adaptation and got better results in experiments. However, the above methods are all based on the research under the stable working condition; without considering the influence of the working condition change of the aircraft engine on the prediction accuracy of RUL under the real flight condition, it still faces the following problems in the practical application process:

1. At present, the prediction models of aircraft engines are all based on a snapshot of flight data, the advantages of real-time monitoring data of aircraft engines are not fully utilized;
2. Because the deep learning network can’t be explained, the RUL prediction model can only be used as a black box, and the problems in the use of aircraft engines can’t be found through the prediction results;
3. In some RUL prediction models, complex signal processing is needed to extract features artificially in advance, which fails to give full play to the features of deep model learning.

In order to effectively provide reliable RUL information for PHM system to better manage the aircraft engines and reduce the maintenance cost, we propose an improved CNN-LSTM aircraft engine RUL prediction method based on convolutional block attention module (CBAM). This method firstly learns all the data in one cycle of aircraft engine by two dimensional convolutional neural network, extracts the potential relationship between feature sequences to generate a feature map, then deduces the attention weight along the two dimensions of spatial and channel by CBAM, and multiplies it with original feature map to adjust the features adaptively. Finally, LSTM has a good learning ability to time series information to predict aircraft engine. Compared with other methods, this RUL prediction model has higher prediction accuracy. In addition, our model can analyze the effect of the original input data on the final prediction result by visualizing the attention weight, and provide suggestions for the use and maintenance of aircraft engines.

Our work is arranged as follows. Section 2 briefed the basic theories needed to build the model described respectively. Section 3 introduced the structure of our RUL prediction model in detail. The experiment is carried out through C-MPASS simulation data, and the experimental results are discussed in Section 4. In Section 5, a summary is given.

## Theoretical methods

### Convolutional Neural Network (CNN)

**Figure 1:**
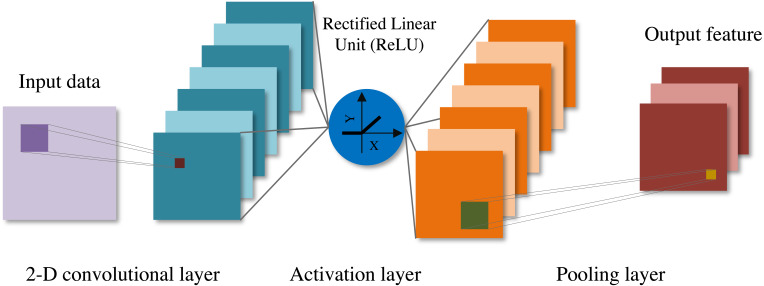
The network structure of convolutional neural network.

CNN is a special multi-layer perceptron (MLP), which has the traits of local connection and weight sharing. Because of its advantages in image processing, it is widely used in computer vision, image classification and other applications (Zhou, 2020). CNN can be divided into one dimensional, two dimensional and three dimensional CNN (1-D CNN, 2-D CNN and 3-D CNN). We mainly use 2-D CNN, which usually includes convolution layer, activation layer and pooling layer. Figure 1 shows a simple convolutional neural network.

The crucial part of CNN is convolution layer. Convolution layer convolves input data through local connection and weight sharing, and its function is to extract features by shifting convolution filters on original data. Convolution filter is also called receptive field. Convolution filter moves along all dimensions of input data, calculates the weight and the dot product of input, and then adds bias to realize convolution operation. The specific convolution operation is:

(1) \begin{eqnarray*}{\mathbf{y}}_{n}={\mathbf{w}}_{m}\ast {\mathbf{x}}_{n}+{\mathbf{b}}_{m}\end{eqnarray*}

where **w**_*m*_ and **b**_*m*_ ∈ *R*^*h*×*w*^ represent the weight and bias of the *m*-th filter, respectively, and their sizes *h* × *w* are specified when designing the network, **x**_*n*_ is the *n*-th region of the input data, and **y**_*n*_ is the convolution output of the filter.

As can be seen from Eq. (1), each convolution filter can generate a corresponding feature map. When multiple filters are specified in a convolution layer, a corresponding number of feature maps can be generated, and the output of the convolution layer can be obtained by stacking all the feature maps in the channel dimension.

The role of activation function is to introduce nonlinear factors to enhance the feature expression ability of the model. At present, the Rectified Linear Unit (ReLU) is the most versatile and effective activation function (Dubey & Chakraborty, 2021), which can improve the network sparsity and reduce the network over-fitting. The expression of ReLU is:

(2) \begin{eqnarray*}ReLU(x)= \left\{ \begin{array}{@{}cc@{}} \displaystyle &\displaystyle x~~if~x\gt 0\\ \displaystyle &\displaystyle 0~~if~x\leq 0 \end{array} \right. \end{eqnarray*}

where *x* is the input of the activation function.

The role of pooling layer is to gradually shrink the feature space size, decrease the amount of parameters of the network and speed up the calculation. The pooling layer is similar to the convolution layer, and it is also performed by filters. The difference is that the filters of the pooling layer do not perform convolution operation on the input data, but perform pooling operation, which is also called downsamples. Generally, the pooling layer can be divided into average pooling layer and max pooling layer. The average pooling layer is to extract the average value from the filter, and The max pooling layer is to extract the max value from the filter. The existing studies indicate that the max pooling layer is preferable in model training (Raj & Kannan, 2022).

### Convolutional block attention module (CBAM)

**Figure 2:**
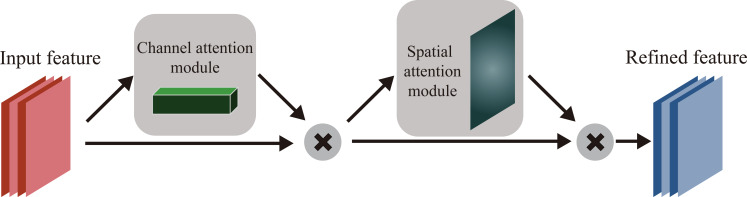
The network structure of CBAM.

Attention mechanism is a data processing method in deep learning, which can learn how different parts of the input affect the output (Chaudhari et al., 2021). CBAM is a kind of attention mechanism which could be used in CNN. CBAM is designed as a simple and competent module to be applied to CNN (Woo et al., 2018). Figure 2 shows the structure of CBAM. CBAM is composed of the Channel Attention Module (CAM) and Spatial Attention Module (SAM). CAM and SAM can perform attention operations in the channel and spatial respectively. This structure design enables CBAM to be quickly combined with existing model, saving parameters and computing resources.

**Figure 3:**
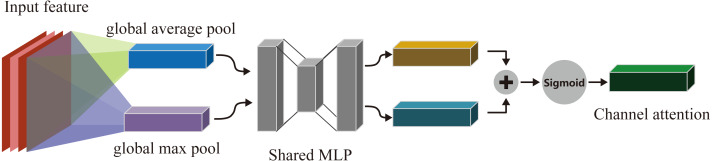
Channel attention module.

As shown in Fig. 3, the CAM firstly performs global average pooling and global max pooling on the input data map **D**(*h* × *w* × *c*) to output two vectors of size 1 × 1 × *c*, and inputs them into two-layer MLP respectively which has *c*/*r* neurons in the first layer ,where *r* is reduction rate and *c* neurons in the second layer. The parameters of these two-layer MLP are shared. Next, add up the outputs of MLP by elements. After that, the result will be activated by sigmoid function to obtain the channel attention **A**_*c*_. Finally, **A**_*c*_ and **D** are multiplied by elements to obtain the weighted output **D**′.**D**′, and the **D**′ is input of SAM.

The channel attention mechanism can be expressed by the formula:

(3) \begin{eqnarray*}{\mathbf{A}}_{c}(\mathbf{D})=\sigma (MLP({\mathbf{P}}_{\mathrm{max}})+MLP({\mathbf{P}}_{avg})) =\sigma {\mathbf{W}}_{1}{\mathbf{W}}_{0}({\mathbf{P}}_{\mathrm{max}})+{\mathbf{W}}_{1}({\mathbf{W}}_{0}({\mathbf{P}}_{avg}))\end{eqnarray*}

where *P*_max_ and *P*_*avg*_ are the results of global max pooling and global average pooling of input features, *σ* is sigmoid activation function, *W*_0_ and *W*_1_ are the weights of the first and second layers of MLP, and their sizes are *c*/*r* × *c* and *c* × *c*/*r* respectively.

**Figure 4:**
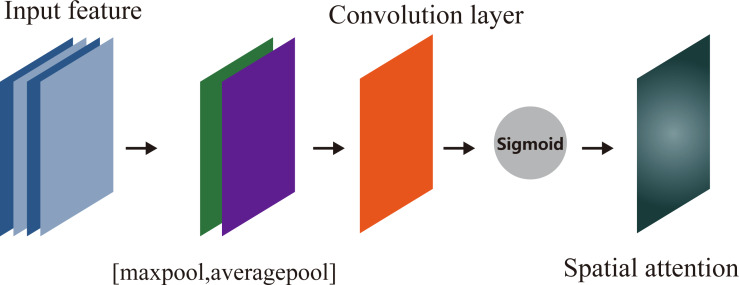
Spatial attention module.

As shown in Fig. 4, the SAM uses the data map **D**′ as input which is the output of CAM, and gets two feature maps with the size of *h* × *w* × 1 through max pooling and average pooling operation in channel, and takes these two feature maps as input of convolution layer containing a filter. And the output is activated by sigmoid to get spatial attention **A**_*s*_. Finally, **D**′ is multiplied with **A**_*s*_ to get the final feature.

The spatial attention can be expressed as:

(4) \begin{eqnarray*}{A}_{s}({\mathbf{D}}^{{^{\prime}}})=\sigma ({f}_{conv}([maxpool({\mathbf{D}}^{{^{\prime}}});avgpool({\mathbf{D}}^{{^{\prime}}})]))\end{eqnarray*}

where *f*_*conv*_ is convolution operation, *maxpool* and *avgpool* are max pooling and average pooling respectively.

### Long and short term memory network (LSTM)

Unlike the general forward feedback network, LSTM is a idiosyncratic kind of recurrent neural network (RNN) (Livieris, Pintelas & Pintelas, 2020). Compared with RNN, LSTM can analyze the input in time series, and it can solve the problem of long-term dependence of the series, avoiding the problems of gradient disappearance and gradient explosion, so it can be used for the analysis of long-term data (Jang et al., 2020).

**Figure 5:**
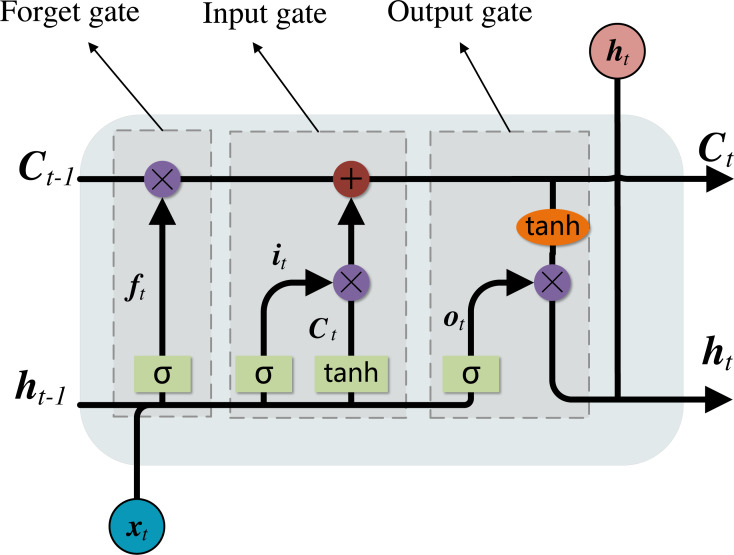
Network structure of LSTM.

Figure 5 shows the internal structure of an LSTM unit which can be mainly divided into three parts: forget gate, input gate, output gate. In each unit, the cell state **C** and the output hidden state **h** will be updated through the 3 internal gates. The forget gate determines the forgotten message **f**_*t*_ according to the input **x**_*t*_ and the previous hidden state **h**_*t*−1_, the input gate selects the candidate memory message ${\widetilde {\mathbf{C}}}_{t}$ through the input control **i**_*t*_ to determine the updated content of the previous state **C**_*t*−1_, and the output gate determines the hidden state **h**_*t*_ that the current cell state passes to the next cell through the output control **o**_*t*_.

The specific calculation process in LSTM network unit is as follows:

(5) \begin{eqnarray*}{\mathbf{f}}_{t}=\sigma ({\mathbf{f}}_{w}\cdot [{\mathbf{h}}_{t-1},{\mathbf{x}}_{t}]+{\mathbf{f}}_{b})\end{eqnarray*}

(6) \begin{eqnarray*}{\mathbf{i}}_{t}=\sigma ({\mathbf{i}}_{w}\cdot [{\mathbf{h}}_{t-1},{\mathbf{x}}_{t}]+{\mathbf{i}}_{b})\end{eqnarray*}

(7) \begin{eqnarray*}{\widetilde {\mathbf{C}}}_{t}=tanh({\mathbf{c}}_{w}\cdot [{\mathbf{h}}_{t-1},{\mathbf{x}}_{t}]+{\mathbf{c}}_{b})\end{eqnarray*}

(8) \begin{eqnarray*}{\mathbf{C}}_{t}={\mathbf{f}}_{t}\odot {\mathbf{C}}_{t-1}+{\mathbf{i}}_{t}\odot {\widetilde {\mathbf{C}}}_{t}\end{eqnarray*}

(9) \begin{eqnarray*}{\mathbf{o}}_{t}=\sigma ({\mathbf{o}}_{w}\cdot [{\mathbf{h}}_{t-1},{\mathbf{x}}_{t}]+{\mathbf{o}}_{b})\end{eqnarray*}

(10) \begin{eqnarray*}{\mathbf{h}}_{t}={\mathbf{o}}_{t}\odot tanh({\mathbf{C}}_{t})\end{eqnarray*}

where **f**_*w*_, **i**_*w*_, **c**_*w*_, **o**_*w*_ are the weights of **f**_*t*_, **i**_*t*_, ${\widetilde {\mathbf{C}}}_{t}$, **o**_*t*_ respectively, and **f**_*b*_, **i**_*b*_, **c**_*b*_, **o**_*b*_ are the biases of **f**_*t*_, **i**_*t*_, ${\widetilde {\mathbf{C}}}_{t}$, **o**_*t*_ respectively, *tanh* is tanh activation function.

## Proposed methodology

**Figure 6:**
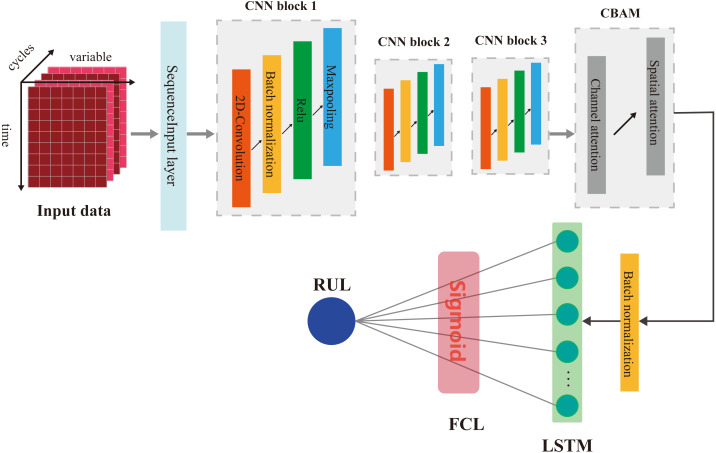
The network structure of the proposed method.

Our proposed RUL prediction model contains a variety of deep learning layers. Figure 6 shows the network structure of our model. First, input data is converted into the data map as shown in Fig. 6, and then the CNN module will extract the features of the input data firstly. In this process, we use three CNN blocks with the same structure. In addition, we introduce the batch normalization layer into the CNN network, its function is to independently normalize the small batch data of all observations in each channel, improve the training speed and effect of CNN and reduce the influence of initialization parameters on training process. Then, the CBAM module infers attention, and carries out adaptive weighting processing. Finally, the obtained feature map is input into LSTM network to mine the hidden relationship between features and service time. Finally, the predicted RUL value is obtained through a fully connected layer and activated by sigmoid function.

**Figure 7:**
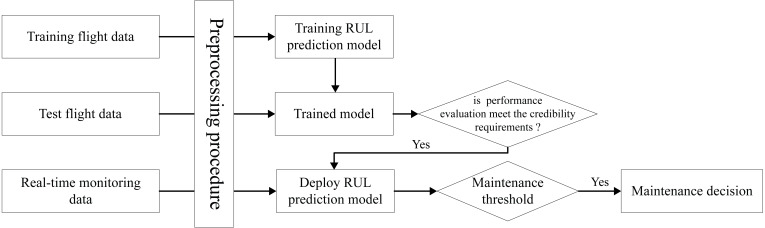
Realization process of aircraft engine RUL prediction with proposed method.

The above is the design process of our model. For the training, verification and deployment of the model, our process is shown in Fig. 7. After the design of our model is completed, the training data set and test data set should be prepare after. Training data is used as input data to train RUL prediction model and get the trained model. After that the trained model will be verified with the test data, and the performance will be evaluated by the specified evaluation method. If the trained model can meet the available standards, we can deploy the model to the aircraft fleet. After each flight, we collect the real-time monitoring data for the same preprocessing, and input it into the RUL prediction model. And then according to the prediction results and the set maintenance threshold, we can make a reasonable decision on engine maintenance, and improve the accuracy of aircraft engine maintenance activities.

## Experimental study

### Dataset description

**Figure 8:**
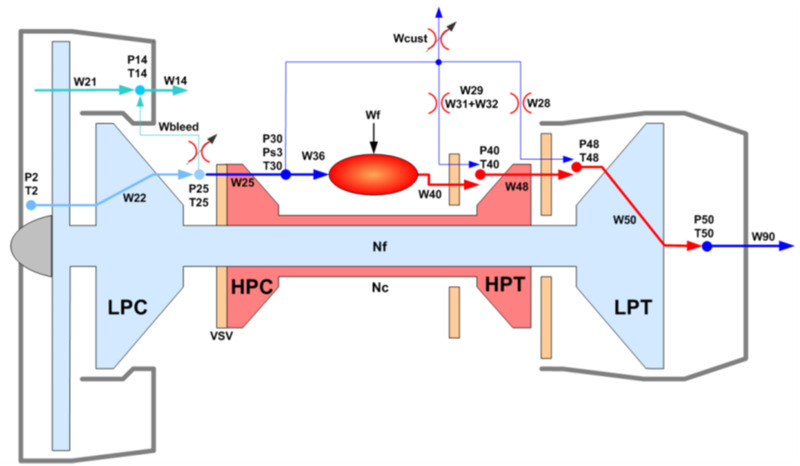
Schematic representation of C-MAPSS model (Chao et al., 2021).

The dataset used in our experiment is the latest Aircraft Engine Run-to-Failure data set published by NASA Ames Research Center in 2020. The dataset was obtained through C-MPASS platform which could simulate the working process of turbofan aircraft engine, its structure is shown in Fig. 8. Compared with the previous run-to-failure trajectories, it considered the performance degradation behavior of the aircraft engine in real flight conditions, and recorded all the flight environment parameters, operation data and degradation data (Chao et al., 2021).

**Figure 9:**
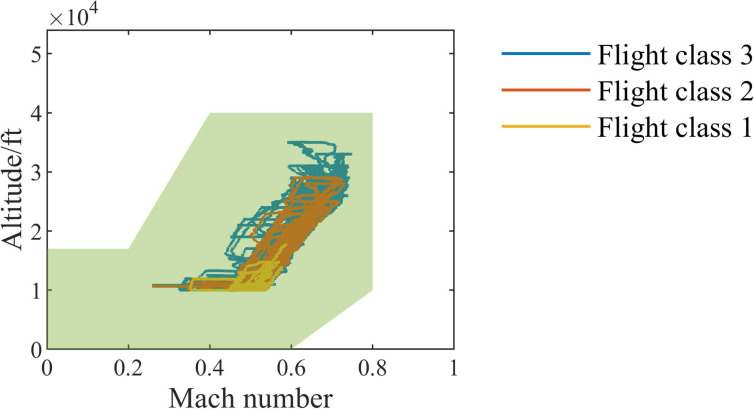
Flight envelope.

In order to compare with other methods, we use *DS*02, the most widely used data set in this data set, which contains the run-to-failure simulation data of nine engines, of which six (unit =2,5,10,16,18,20) are the training set data and three (unit =11, 14, 15) are the test set data. The recorded data of each engine contains four scenario descriptors (*W*), 13 measured physical properties (*X*_*s*_), 18 virtual sensors data (*X*_*v*_) and 10 model health parameters (*θ*). Table 1 shows the description of monitoring parameters. The units are divided into three flight classes (Flight class 1, Flight class 2, and Flight class 3) according to the length of operation time (short-length flights, medium-length flights, and long-length flights). Figure 9 shows the working conditions of different flight classes of engines in the flight envelope, and the green area in the figure is the flight envelope of the engine.

**Table 1:** The description of aircraft engine measurement data.

	Symbol	Description	Units
1	alt	Altitude	ft
2	Mach	Flight Mach number	–
3	TRA	Throttle-resolver angle	%
4	T2	Total temperature at fan inlet	° R
5	Wf	Fuel flow	pps
6	Nf	Physical fan speed	rpm
7	Nc	Physical core speed	rpm
8	T24	Total temperature at LPC outlet	° R
9	T30	Total temperature at HPC outlet	° R
10	T48	Total temperature at HPT outlet	° R
11	T50	Total temperature at LPT outlet	° R
12	P15	Total pressure in bypass-duct	psia
13	P21	Total pressure at fan outlet	psia
14	P24	Total pressure at LPC outlet	psia
15	Ps30	Static pressure at HPC outlet	psia
16	P40	Total pressure at burner outlet	psia
17	P50	Total pressure at LPT outlet	psia
18	T40	Total temp. at burner outlet	° R
19	P30	Total pressure at HPC outlet	psia
20	P45	Total pressure at HPT outlet	psia
21	W21	Fan flow	pps
22	W22	Flow out of LPC	lbm/s
23	W25	Flow into HPC	lbm/s
24	W31	HPT coolant bleed	lbm/s
25	W32	HPT coolant bleed	lbm/s
26	W48	Flow out of HPT	lbm/s
27	W50	Flow out of LPT	lbm/s
28	epr	Engine pressure ratio (P50/P2)	–
29	SmFan	Fan stall margin	–
30	SmLPC	LPC stall margin	–
31	SmHPC	HPC stall margin	–
32	NRf	Corrected fan speed	rpm
33	NRc	Corrected core speed	rpm
34	PCNfR	Percent corrected fan speed	pct
35	phi	Ratio of fuel flow to Ps30	pps/psi
36	fan_eff_mod	Fan efficiency modifier	–
37	fan_flow_mod	Fan flow modifier	–
38	LPC_eff_mod	LPC efficiency modifier	–
39	LPC_flow_mod	LPC flow modifier	–
40	HPC_eff_mod	HPC efficiency modifier	–
41	HPC_flow_mod	HPC flow modifier	–
42	HPT_eff_mod	HPT efficiency modifier	–
43	HPT_flow_mod	HPT flow modifier	–
44	LPT_eff_mod	LPT efficiency modifier	–
45	LPT_flow_mod	HPT flow modifier	–

**Figure 10:**
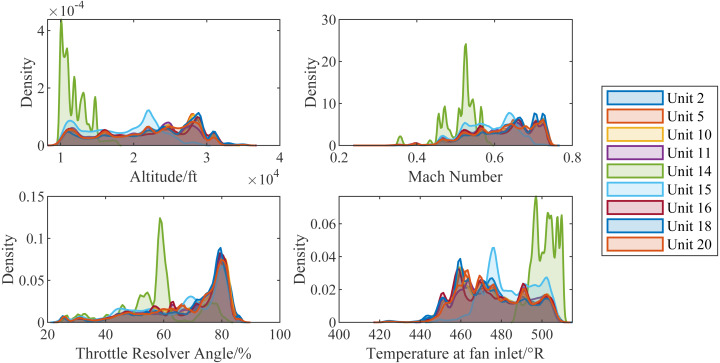
Kernel density estimations of scenario descriptors of different aircraft engines.

Figure 10 shows the kernel density estimations of scenario descriptors (*W*) of each engine. From the distribution, we can see that the working environment of different engines is different. Especially, compared with other engines, the working altitude of unit14 is lower. Under this more realistic condition, the RUL of the aircraft engine is harder to predict.

### Experimental process

#### Data preprocessing

The preprocessing of the monitoring data includes slicing and normalization. Slicing refers to selecting a part of the recorded data of the aircraft engine as the input data of RUL prediction. This is because the monitoring data of aircraft engine in one service cycle contains a large amount of repeated and redundant information. We divide it into 400 copies equally according to the total time in one service cycle, and the data of the corresponding time is extracted and recombined into input data, which can ensure that the input data can describe the RUL information of the aircraft engine, reduce the computing resources and improve the learning speed.

In order to eliminate the training error caused by different measuring units of monitoring data, we normalize the data monitored by different sensors. The normalization method adopted in this article is Min-Max normalization, and the calculation formula is:

(11) \begin{eqnarray*}\widehat{d}= \frac{d-{d}_{min}}{{d}_{max}-{d}_{min}} \end{eqnarray*}

*d*_*max*_, *d*_*min*_ are the maximum and minimum value of the variable, and *d*, $\widehat{d}$ are the original and normalized values of the variable.

#### Evaluation metric

In order to quantify the performance of our proposed RUL prediction model, we select the *RMSE* and *Score* to measure the prediction accuracy of the model (Custode et al., 2022).

*RMSE* is the root mean square error, which is often used as a measure of error in deep learning models, and can reflect the overall deviation between the predicted RUL and true RUL. It can be expressed as following formula:

(12) \begin{eqnarray*}RMSE=\sqrt{ \frac{1}{n} \sum _{i=1}^{n}({R}_{p}^{i}-{R}_{t}^{i})^{2}}\end{eqnarray*}

where *R*_*p*^*i*^_ is the *i*th engine predicted value of RUL , *R*_*t*^*i*^_ is the *i*th engine true value of RUL, and *n* is the number of predicted RUL.

*Score* function is an evaluation function designed by the data set provider. The lower the score, the better the prediction effect of the model. Its mathematical expression is:

(13) \begin{eqnarray*}Score= \left\{ \begin{array}{@{}cc@{}} \displaystyle &\displaystyle \sum _{i=1}^{n}\text{exp}(- \frac{{R}_{p}^{i}-{R}_{t}^{i}}{13} )-1~~if~({R}_{p}^{i}-{R}_{t}^{i}\leq 0)\\ \displaystyle &\displaystyle \sum _{i=1}^{n}\text{exp}( \frac{{R}_{p}^{i}-{R}_{t}^{i}}{10} )-1~~if~({R}_{p}^{i}-{R}_{t}^{i}\gt 0) \end{array}. \right. \end{eqnarray*}

#### Parameter settings of the model

In our prediction model, some parameters of network layers need to manually set, *e.g.*, the size and number of filters in convolution layer. For C-MPASS data set, the parameter settings of some network layers in our RUL prediction model are shown in Table 2. Under this setting, there are about 3 × 10^6^ learnable parameters in our network.

As for the setting of training options, we set the number of iterations to 400, optimize the network parameters by using the adaptive moment estimation (Adam) optimization algorithm, and the batch size is six, the initial learning rate is 0.01, and set the gradient threshold to two to prevent the gradient explosion.

### Experimental results and analysis

#### Comparison and discussion of prediction results

**Figure 11:**
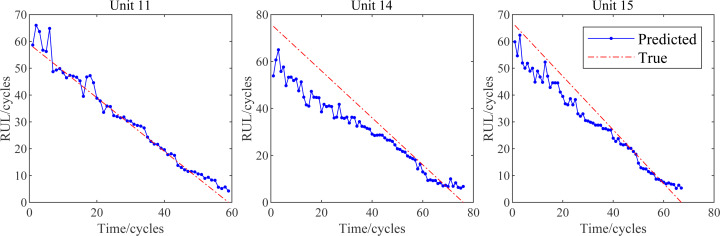
RUL prediction result of test data set.

After the training process, input the test set data into the trained model and get the predicted RUL values. Figure 11 shows our RUL prediction results. As shown in Fig. 11, the predicted RUL values of our RUL prediction model are generally distributed near the true values during the whole aircraft engine service life. Therefore, our model can be applied to predict aircraft engine RUL, and the prediction results can provide reliable suggestions for the use and maintenance of aircraft engines.

**Table 2:** RUL prediction model parameter setting.

**Layer name**	**Parameters**
SequenceInput Layer	**size:** (400,42,1)
Convolution_2d layer 1	**filter size:** (10, 3); **number of filters:** 10; **step size:** (10, 3)
Maxpooling_2d Layer 1	**filter size:** (5, 5); **padding:** same
Convolution_2d layer 2	**filter size:** (3, 3); **number of filters:** 10
Maxpooling_2d Layer 2	**filter size:** (5, 5); **padding:** same
Convolution_2d layer 3	**filter size:** (5, 5); **number of filters:** 10
Maxpooling_2d Layer 3	**filter size:** (5, 5); **padding:** same
Channel attention Layer	**number of channels:** 10; **reduction rate:** 2
Spatial attention Layer	**filter size:** (3, 3)
Lstm Layer	**number of hidden units:** 128
FullyConnected Layer	**output size:** 1

After the RUL prediction result is obtained, the result is evaluated by the evaluation function provided previously to quantify the model performance. In addition, we also use MLP, FNN, CNN and CNN-LSTM (Kong et al., 2019) to predict the test data set. The comparison results are shown in Table 3. Judging from the comparison results in the table, our improved CNN-LSTM based on CBAM prediction model has better performance. Its Score is improved by about 25.9% and its RMSE is reduced by 17.4% compared with other best methods.

**Table 3:** Performance comparison with other neural network methods.

**Method**	**RMSE**	**Score (×10^5^)**
MLP	8.34	13.43
FNN	7.89	9.21
CNN	7.14	7.60
CNN-LSTM	6.66	7.80
**Ours**	**5.50**	**5.78**

#### Analysis of prediction process

**Figure 12:**
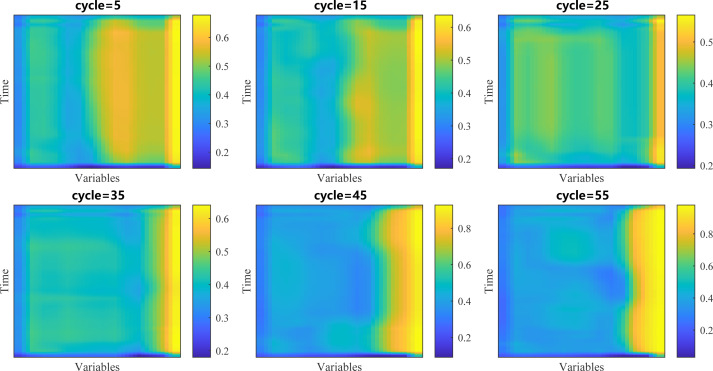
Visualization of spatial attention.

In the process of RUL prediction, different variables have different influences on the prediction results in the input data map, and this difference can be described by CBAM’s spatial attention in our model. Therefore, we extracted the spatial attention matrix in the 5th, 15th, 25th, 35th, 45th and 55th cycles of unit 11, mapped it into the size of the input data, and the result is displayed in the form of the hot map in Fig. 12.

**Figure 13:**
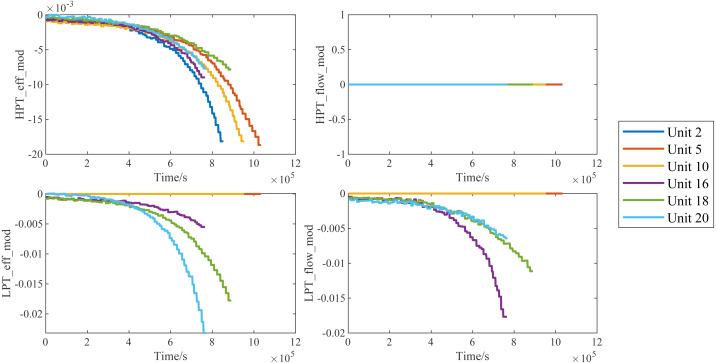
Traces of the degradation imposed on the the four variables.

In the figure, we are able to distinctly see that the weights of different variables are obviously different. A larger weight means that the network will focus more on the changes of the corresponding variables in RUL prediction, while a smaller weight means the opposite. We found four variables with the largest weights, namely *HPT*_*eff*_*mod*, *HPT*_*flow*_*mod*, *LPT*_*eff*_*mod* and *LPT*_*flow*_*mod*, and showed the changes of their values with the service time in Fig. 13. The results show that in the whole life cycle, the changes of the values of most variables are strongly correlated with the service time, so their values also reflect the health of aircraft engines to some extent.

## Conclusions

This article presents an enhanced RUL prediction method of aircraft engine based on CBAM. It mainly combines CBAM attention mechanism in the CNN-LSTM network. The experimental verification is carried out by C-MPASS engine degradation data set, and the following conclusions are obtained:

1. The CNN-LSTM network with attention mechanism has better feature extraction ability and time series analysis ability. Compared with the existing methods, it can analyze all the data of aircraft engine running under different working conditions. The experimental results indicate that the performance of the proposed prediction model is good, and most of predicted RUL values are near the true RUL values, which has high feasibility. Compared with the current methods with high prediction accuracy, the RMSE of our method decreased by 17.4%, and the score of the prediction model was improved by 25.9%;
2. CBAM module can effectively find the key variables that reflect the health condition of aircraft engine. By paying more attention to these key variables, the CNN-LSTM network can have better prediction accuracy. Furthermore, CBAM makes the model interpretable to some extent, which can provide some help for sensor arrangement and troubleshooting of aircraft engines;
3. Our proposed RUL prediction method doesn’t involve any analysis on aircraft engine composition, fault mechanism, *etc.*, so the model can be deployed and applied quickly. The RUL information provided by our method can assist the maintenance management system to make decisions, in order to ensure the reliability of the aircraft engine and reduce the maintenance cost.

## Supplemental Information

Supplemental Information 1 Code All codes run in the environment of MATLAB2021a and higher.

### Additional Information and Declarations

#### Data Availability

The following information was supplied regarding data availability: The data is available at figshare: Li, Hao (2022): N-CMAPSS_DS02-006.h5. figshare. Dataset. https://doi.org/10.6084/m9.figshare.20436504.v1. The code is available at GitHub: https://github.com/HaoLi1998/RUL-prediction-model.git.
